# A Novel Strengthening Method for Damaged Pipeline under High Temperature Using Inorganic Insulation Material and Carbon Fiber Reinforced Plastic Composite Material

**DOI:** 10.3390/ma12213484

**Published:** 2019-10-24

**Authors:** Yeou-Fong Li, Tsung-Han Tsai, Tzu-Hsien Yang

**Affiliations:** 1 Department of Civil Engineering, National Taipei University of Technology, 1, Sec. 3, Chung-Hsiao E. Rd., Taipei 10608, Taiwan,; yfli@mail.ntut.edu.tw (Y.-F.L.); henryetsong@gmail.com (T.-H.T.); 2 Department of Materials and Mineral Resources Engineering, National Taipei University of Technology, 1, Sec. 3, Chung-Hsiao E. Rd., Taipei 10608, Taiwan

**Keywords:** perlite, vermiculite, insulation material, carbon fiber reinforced plastic, strengthening method

## Abstract

In this paper, a strengthening method for the damaged high-temperature steel pipeline using inorganic insulation material which was confined by carbon fiber reinforcement plastic (CFRP) composite materials was proposed. Two inorganic insulation materials were composed of magnesium phosphate cement (MPC) mixing with perlite and vermiculite powders, respectively. The influences of insulation material composites with various ratios of the perlite or vermiculite powder were discussed, in terms of compressive strength and thermal conductivity coefficients of inorganic insulation materials. The insulation materials confined by carbon fiber reinforced polymer jackets for enhancing the mechanical behavior were also investigated. From the experimental results, the main finding of the work was that the inorganic insulation materials added to the perlite powder represented greater insulation capability than added vermiculite ones under the condition of the same compressive strength. Different ratios of perlite inorganic insulation material cylinders with the dimension of ϕ 10 cm × 20 cm were confined by one layer and two layers of CFRP composite material. The compressive strength of the specimens increased by 258%–927% after using 1-layer CFRP composite material and increased by 480%–1541% after applying 2-layer CFRP composite material. A peak strength prediction model of insulation materials confined by CFRP was proposed, and it was found that the proposed model accurately predicted the peak strength of the inorganic insulation material cylinder. Finally, a verification test of the strengthening method for damaged high-temperature pipeline was performed to prove that the proposed strengthening method is feasible.

## 1. Introduction

The petrochemical industry has been developing for many decades. The pipelines in the petrochemical factories operate in high-temperature environments and pose a danger as deterioration led to thinning of the thickness of the pipelines. Damages to the pipeline used in a high temperature decrease its resistance for high internal pressures and caused noticeable accidents such as an explosion or severe fracture. At present, there are many new materials and innovative methods to improve corrosion resistance and enhance thermal insulation works for pipelines, such as rock wool coated pipeline and spray coating on the surface of the pipeline. The rock wool is a thermal insulator widely used in the industry. However, water intrusion into the insulation system through the interface of the metal and rock wool is inevitable because these joints cannot be made water-tight using adhesives or sealants. When water penetrates through the system and the rock wool cannot inhibit water intrusion among the interface, crevice and pitting corrosion of the interior surface of the metallic pipeline can occur [1].

The current strengthening methods for coating thermal insulation material on the surface of the pipeline still have issues to overcome, especially, when the corroded pipeline is installed in the high-temperature environment. In general, the insulation materials for pipeline thermal insulation works have a mostly porous morphology, because the porous materials have an advantage of the excellent thermal insulation properties. Porous materials employed as the heat insulation barrier on the surface of the pipeline construction cannot enhance its reinforcement simultaneously at the same time. However, for consideration of its lightweight design, the excessive weigh-lighted porous material used in pipeline that cannot bear the high internal pressure due to the corroded pipeline with lower strength. Especially, after the corroded pipeline used in the high-temperature environment led to the damage condition, the severe corrosion diminished the capability of the pipeline to operate, causing the emergency accident. Therefore, the thermal insulation layer engineering of the pipeline firstly wraps a protective layer around the surface of an unused pipeline before placing it in the high-temperature surroundings. A waterproof material was coated on the pipeline as the first step of the protective construction, and then covered the porous materials as the thermal insulation layer for a thermal barrier.

In this study, a strengthening method for the high-temperature steel pipeline was proposed by using inorganic insulation materials confined by carbon fiber reinforcement plastics (CFRP) composite materials. Recently, the price of carbon fiber is much less than that in last two decades; therefore, the carbon fiber was widely used in sporting goods, automotive, aerospace, and civil engineering. CFRP composite material has features of acid and alkali resistance, anti-corrosion, and high strength-to-weight ratio [2]. CFRP composite material can significantly enhance the compressive strength of the pipeline and prevent leakage of the material inside the pipeline. 

This study collected kinds of literature related to the thermal conductivity coefficient and the effect of different additives to concrete under high-temperature conditions. Moreover, the concrete cylinders confined with CFRP composite material were investigated as follows. The vermiculite and expanded perlite as aggregates were placed in lightweight concrete, shotcrete, and clay wall brick, and found the thermal conductivity decreased with increasing content of the expanded perlite. Moreover, the increase in the content of expanded perlite also decreased the modulus of elasticity and compressive strength, whereas the modulus of elasticity dropped more dramatically than the decrease of compressive strength [3,4,5,6,7,8]. The rock wool powder, furnace slag, and fly ash were used to replace the partial content of the cement, then the fluidity, compressive strength, and some mechanical properties of the composites were discussed [9]. In the meantime, the expanded polystyrene was used to replace natural aggregates in concrete, and the thermal conductivity was discussed [10]. Different types of light-weight fine aggregates, micro hollow glass spheres, cannabis fiber, rock, and palm shell oil foamed as aggregates of concrete through the experiment to study the effect of various water and aggregate contents on the thermal conductivity properties [11,12,13,14]. Garcia *et al.* aim to investigate how nanofibers affect the dynamic behavior and delamination resistance of glass fiber reinforced polymer (GFRP) composites. Experiments and numerical simulations using finite element modeling (FEM) analysis are used to estimate the natural frequencies, the damping ratio, and inter-laminar strength in GFRP composites [15,16].

Most equations to predict peak strength for concrete cylinder confined by FRP composite materials were from the experimental results and empirical curve fitting [17,18,19,20]. Moreover, Li *et al.* adopted the Mohr–Columb failure envelope theory to propose a peak strength of the constitutive model for concrete cylinder confined by CFRP composite material. The strain at the peak strength was obtained from the regression analysis of the experimental results. A second-order polynomial equation was used to present the stress-strain curve of the constitutive model [21].

The strength of FRP confined concrete under normal and high-temperature usages were tested. The results showed that the strength deeply reduced at high temperature, and FRP composite material cannot resist the high-temperature environment [22]. Mattos et al. studied the applicability of GFRP composites to repair rusted metallic pipelines under a temperature of about 60–90 °C. The 70% loss of the inner cross-sectional pipeline was repaired with GFRP composite material. It was found that the damage pressure of the test results and analytical results was close to each other [23].

## 2. The Proposed Strengthening Method

**Figure 1:**
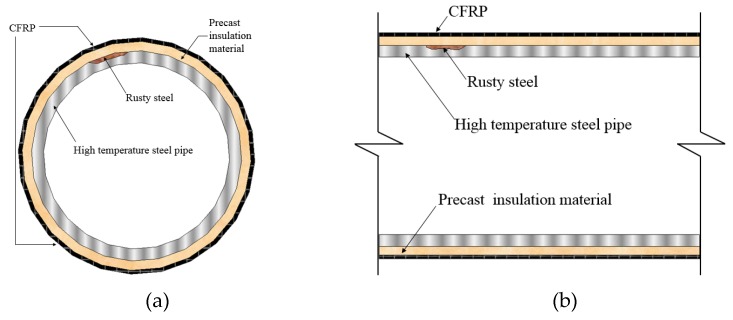
The proposed strengthening method for high-temperature steel pipe. (**a**) Lateral cross-section; (**b**) Longitudinal cross-section.

**Figure 2:**
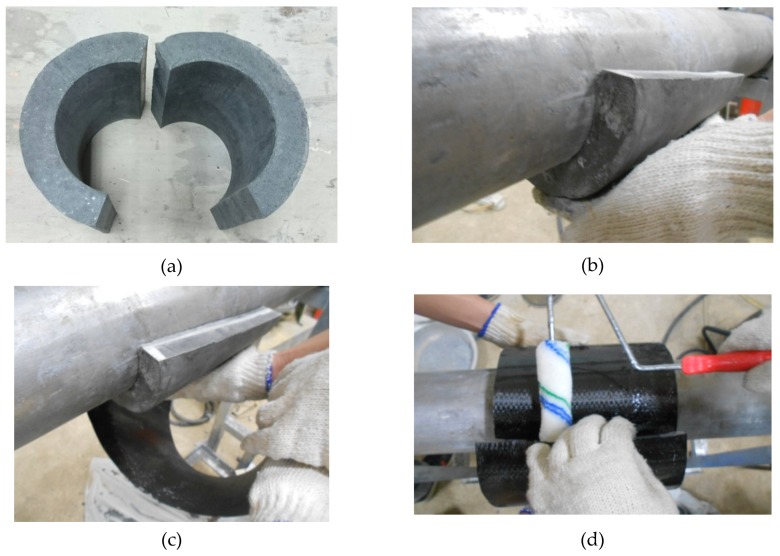
The procedure scheme for rehabilitation method of the high-temperature used pipeline. (**a**) Precast C-shaped insulation material; (**b**) The pipeline was covered by two C-shaped precast insulation material; (**c**) The C-shaped insulation material was wrapped by carbon fiber reinforcement plastic (CFRP) jacket; (**d**) Epoxy resin was used as an adhesive to fasten the CFRP jacket.

The illustration of the proposed strengthening method for the high-temperature steel pipeline was shown in Figure 1. Two precast C-type insulating materials were placed on the upper and lower portions or the left and right portions of the pipeline. Figure 2 shows the procedures of the strengthening method for emergency maintenance of the damaged pipeline in a high-temperature condition. First, the outer surface of the high-temperature pipeline was covered by two C-shaped precast insulation materials, and then the C-shaped precast insulation materials were wrapped by 1-layer CFRP composite material jacket. Finally, epoxy resin was used to affix the carbon fiber jacket to avoid the unexpected slippage of the interface between carbon fiber and the insulation material layer.

Epoxy type had a significant effect on the performance of samples regarding their resistance to corrosion. The precast C-type inorganic insulation material and CFRP composite material with resin epoxy can provide an excellent thermal insulation and water resistance against the corrosion of interior pipeline. Typically, the glass transition temperature of the epoxy resin is about 120 °C. However, epoxy resin used in the CFRP composite material degrades in the high-temperature environment, because the high-temperature thermal condition can break down its bonding strength at the interface of CFRP and the pipeline. In order to decrease the impact of the thermal condition on the CFRP composite material, this study focused on the effects of the different additive with magnesium phosphate cement (MPC) on its thermal conductivity coefficient and the compressive strength of the pipelines used in the high-temperature surroundings. 

## 3. Insulation Materials and Properties

### 3.1. Materials

The perlite and vermiculite powders as admixtures for thermal insulation and were mixed into the MPC. The component of MPC mainly consisted of magnesium oxide while the component of the cement was primarily silicon dioxide. The specific feature of the MPC has rapid solidification characteristics. There was only a working period of 10–15 min to the initial setting of solidification of the MPC, and the strength of the sample enhanced due to high early strength during the period of the initial curing. The specimen with the perlite and vermiculite powder of 0, 5, 10, 15, 20, and 25 wt% for the compressive strength and solid thermal conductivity tests. The description of the various specimens conditions is shown in Table 1. Three specimens were made for each additional ratio of the additive. A total of 33 specimens, including 3 benchmark specimens, experimented for the compression test and thermal conductivity test, respectively.

The material mechanical properties of carbon fiber sheet and epoxy resin were shown in Table 2. Epoxy polymer on the carbon fiber was used for two applications. First one was for resining the precast of elastic CFRP jacket, and the second one was for adhering the overlap of the precast carbon-fiber jacket to the C-shaped thermal insulation layer.

### 3.2. Compressive Strength and Thermal Conductivity Tests

**Figure 3:**
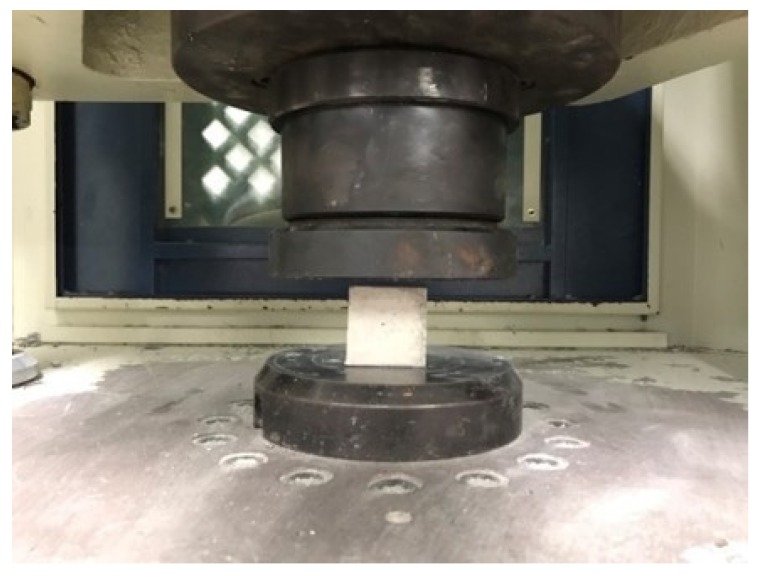
The illustration photo of compression test for cubic specimen.

The compressive strength test of the specimen with the dimensional aspects of 5 mm × 5 mm × 5 mm standard cubic specimens performed according to ASTM C109/C M109-02 [24]. This test program was undertaken by the 100 tf universal-testing machine at the material laboratory of the Department of Civil Engineering, National Taipei University of Technology. The illustration photo of compression test for cubic specimen is shown in Figure 3.

For the solid thermal conductivity of the material, the Fourier law was adopted, which is, the heat flux is proportional to the temperature gradient, as shown in Equation (1).

(1) $$\frac{dQ}{dt}=-kA\nabla T$$

where,

$\frac{dQ}{dt}$: Heat flux (Unit: W)
A: Heat flow through the cross-sectional area (Unit: m^2^)
k: Thermal conductivity coefficient (Unit: W/(m°C))
∇T: Temperature gradient along heat flow direction (Unit: °C/m)

**Figure 4:**
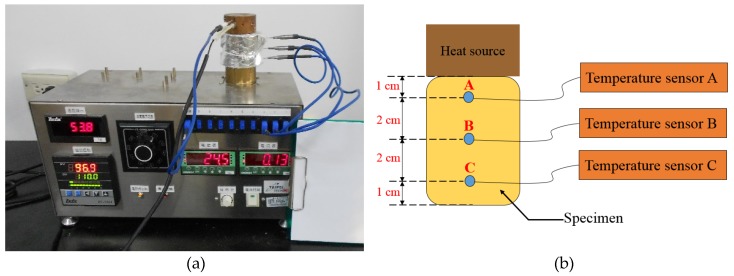
Illustration of the thermal conductivity test (SUS304, Xiwnag Jiazu LTD., Kaohsiung, Taiwan). (**a**) Thermal conductivity test setup; (**b**) Thermal conductivity test specimen.

The thermal conductivity test specimen is a cylindrical body of diameter of 5.6 cm and length of 6 cm for the thermal conductivity coefficient test according to the specifications of ASTM E1225-13 [25]. The electric bar as a heating source provided the thermal source of the specimen above the copper block, as shown in Figure 4a. The thermal grease was pasted on the top and bottom sides of the specimen, the contact thermal resistance at the interface of the copper block. Three contact holes were constructed with the interval distance from the heat source of 1 cm, 3 cm, and 5 cm, respectively, as shown in Figure 4b. The temperature measurement was conducted using Thermocouple T-type class. This test was assumed as only one dimension of the heat flow, and the thermal conductivity of the material was calculated according to Equation (1).

## 4. The Test Results of Insulation Materials

### 4.1. The Compression Test Results

**Figure 5:**
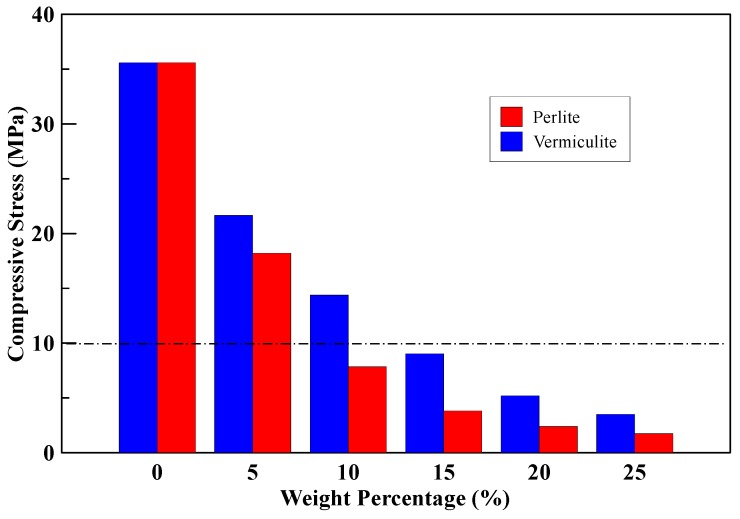
The relationship diagram between compressive strength and the content of perlite or vermiculite (by percentage weight) mixed with magnesium phosphate cement (MPC).

The specimen for the compression test was made from the MPC mixed with perlite or vermiculite and formed into a test block of size 5 cm × 5 cm × 5 cm. The ratio of water to MPC was 0.22, and the compressive strength was tested by the universal testing machine after the ages of 3 days. The compressive strength of the specimens with different amounts of perlite and vermiculite mixed MPC is shown in Table 3. The compressive strength of the specimens decreased as the content of the perlite or vermiculite powder increased, no matter what the additive of the perlite or vermiculite powder mixed with MPC. Decrease of the compressive strength was more obvious with increasing the additive of perlite than increasing the additive of vermiculite mixed with the MPC. To increase weight percentage of the perlite and vermiculite powders from 0% to 25% added in the concrete block can decrease the compressive strength as shown in Figure 5. Though perlite powder has weak compressive strength, the following thermal conductivity test showed it had a good thermal insulation due to its porous structure.

### 4.2. The Thermal Conductivity Test Results

Porosity of additive such as perlite is one of the factors affecting the thermal conductivity of concrete. Enclosed pores reduce the conductivity due to low thermal conductivity of air. Replacing normal aggregate with the expanded perlite, therefore, increases the total porosity of concrete which affects the thermal conductivity [26]. 

**Figure 6:**
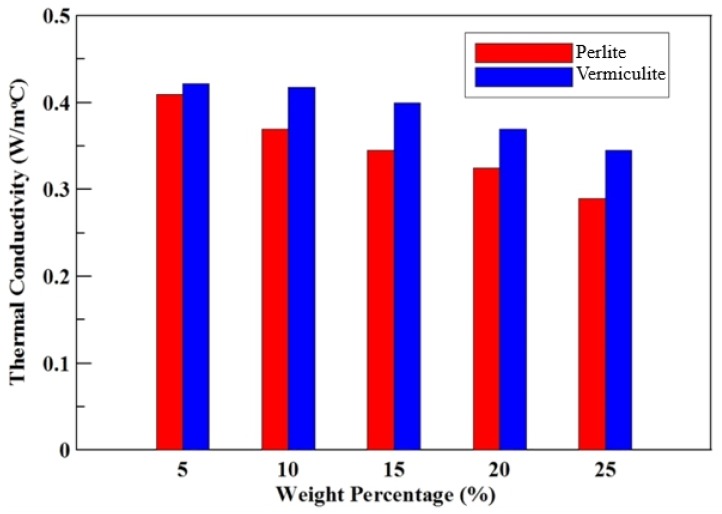
The relationship diagram between conductivity coefficient and the content of perlite or vermiculite (by percentage weight) mixed with MPC.

The thermal conductivity coefficient of the specimens with different composition of the perlite and vermiculite powders, respectively, mix with the MPC is shown in Table 4. The thermal conductivity coefficient of the specimen decreased while the additional content of perlite or vermiculite mixed in the specimen increased. The specimen of lower thermal conductivity coefficient means that heat insulation layer can provide a thermal barrier. When the amount of the additive increased, the thermal conductivity coefficient of the specimen with MPC mixing of the perlite decreased significantly more than that of the specimen with MPC mixing of vermiculite. The thermal conductivity coefficients of the specimen with MPC mixing of the perlite and vermiculite were shown in Figure 6. 

According to Figure 5 and Figure 6, the effect of the different additives in the specimens related to their compressive strength and thermal conductivity coefficient. Wongkeo et al. [27] used bottom ash to replace cement in concrete, with a weight ratio of 0%, 10%, 20%, and 30%. When substituted to 10%, the compressive strength was 10.1 MPa. The experiment was based on the compressive strength of 10 MPa as a criterion and obtained the optimum amounts of perlite and vermiculite additives of 8% and 13% by an interpolation method, refer to Figure 5. 

The results showed the compressive strength of the specimen mixed with the vermiculite powder was higher than that of the specimen mixed with the perlite powder. On the contrary, the additional vermiculite powder in the specimen did not enhance its thermal insulation effect. The flow rate of the slurry by adding perlite powder was higher than that with additional vermiculite powder. Therefore, perlite powder was chosen to mix with the MPC because of good thermal insulation and workability. After accomplishing the thermal conductivity test and the compressive strength test of the thermal insulation layer relied on different composition parameters of the perlite and vermiculite powders, the compressive strength of the perlite powder mixed with the MPC cylindrical specimen confined by CFRP composite material will be discussed in the next section. 

## 5. The Compression Test of CFRP Confinement

A review of earlier literature indicated that the existing constitutive models for confined concrete were proposed for high strength. However, the porous insulation material is a material of low strength and few studies investigated the uniaxial compression test on low strength material, such as porous insulation mortar confined by CFRP jacket. This study proposed a novel strengthening method for the high-temperature pipeline was to cover two C-shaped precast inorganic insulation layers on it. Then, the precast CFRP jacket was wrapped to confine the inorganic insulation layers.

### 5.1. Specimen Preparation and Experimental Setup

The thin layer of primer epoxy applied to the surface of the mortar cylinder. After the primer epoxy resin on the surface of the mortar was cured, the carbon fiber sheet was wrapped on the surface of the insulation material cylinders. For each layer of carbon fiber sheet, epoxy resin was pasted by using a painting brush to deeply immerse the carbon fiber. The extra epoxy for each layer was squeezed out using a flat plastic scraper. 

As seen in Figure 5, if the compressive strength of the specimen with the insulation material reaches to 10 MPa, the content of the insulation material mixed with perlite is about 8%. The appropriate perlite ratios were 8%, 15%, 20%, and 25%, and the dimensional was ϕ10 cm × 20 cm. Each of the insulation material cylinders was wrapped with additional 1-layer and 2-layer CFRP composite material. For each experimental parameter of the perlite additive, three insulation material cylinders were made. The three cylinders without CFRP were used as the reference (benchmark). A total of 36 insulation material cylinders were tested; the controlled variables of the cylinders are the perlite ratio, and the number of layers of CFRP, as shown in Table 5. The notation of the specimen showed as followings. The first letter “P” stood for perlite, the following number stood for the percent ratio of the perlite; the second letter “C” stood for the CFRP confinement, and the following number was the amounts of the layers of CFRP composite material.

**Figure 7:**
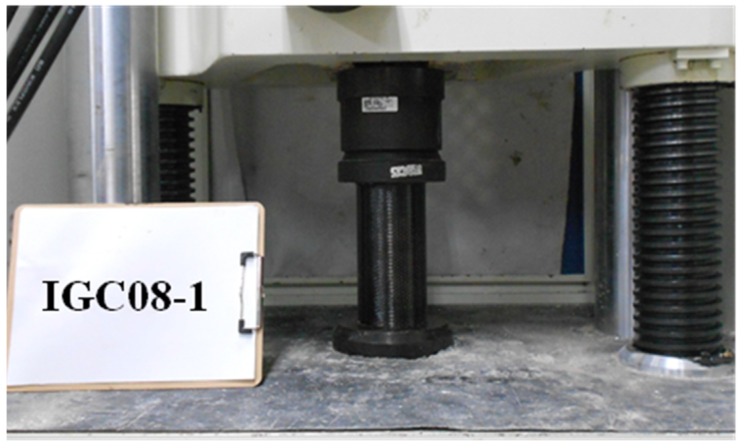
The illustration photo of compression test for cylindrical specimen.

This test program was undertaken by the 100 tf universal-testing machine at the material laboratory of the Department of Civil Engineering, National Taipei University of Technology. The experimental equipment included a load cell, a linear voltage displacement transformer, and an analog/digital converter with a signal amplifier, and a personal computer, shown in Figure 7. In order to ensure that the uniaxial force was applied uniformly on the top and bottom surfaces of the mortar cylinder, two surfaces of the specimen were covered horizontally by gypsum paste. Loading rate of the actuator was 1 mm/sec, and the loading process stopped when the axial load began to decrease. 

### 5.2. Compression Test Results 

**Figure 8:**
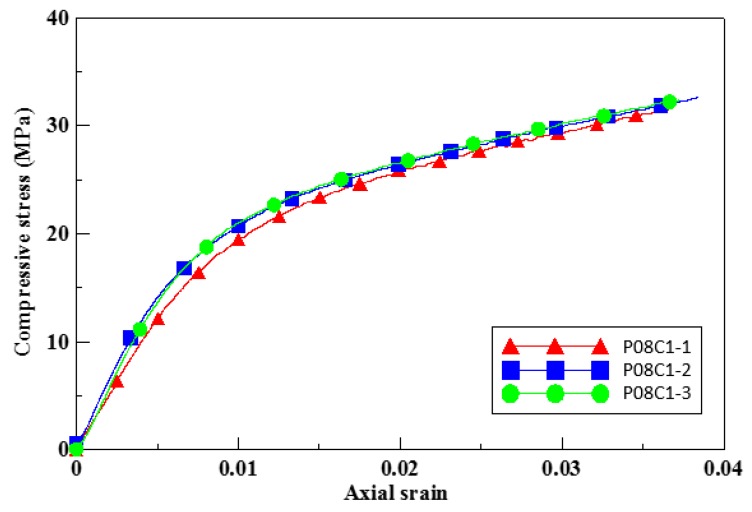
The illustration photo of compression test for cylindrical specimens P08C1.

**Figure 9:**
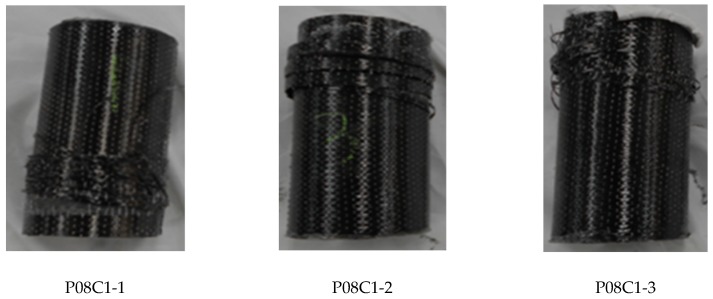
The failure photos of specimens P08C1.

Three strain gauges were mounted on the top, middle, and bottom of each insulation material cylinder wrapped with CFRP composite material; and the lateral strains of the insulation material cylinder were measured. Since the deformation of the insulation material cylinder was not uniform, the measured strains exhibited variability. Figure 8 is the stress–strain curves of the specimens P08C1, when the stress reaches 16 MPa, the stiffness begins to decrease. When the stress reaches ultimate strength, the specimen produced a huge burst sound when on its failure. The failure photos of specimen P08C1 were shown in Figure 9. 

The compressive strength of the specimen with insulation materials attached after applying the strengthening layer rehabilitated by CFRP is shown in Table 6. The compressive strength of the specimens P08, P15, P20, and P25 was increased by 258%–927% after confined by 1-layer CFRP composite material, and that of the specimens P08, P15, P20, and P25 was increased by 480%–1541% after confined by 2-layer CFRP composite material. The results showed that the compressive strength of the specimen with the insulation materials confined by CFRP composite materials can effectively increase.

### 5.3. Proposed Peak Stress Formula

The peak stress formula of insulation material confined by CFRP was adopted according to the constitutive model proposed by Li et al. [19]. The physical-based constitutive model for the confined strength of the specimen with the insulation materials ($f_{cc}^{’}$) can be expressed as follows:

(2) $$f_{cc}^{’}=f_{co}^{’}+f_{l}^{’}\tan^{2}\left(45^{0}+\frac{\phi }{2}\right)\;(\mathrm{Unit}:\;\mathrm{MPa})$$

where

(3) $$f_{l}^{’}=k_{c}\frac{2\times n\times t\times E_{cf}\times \varepsilon_{cf}}{D}\;(\mathrm{Unit}:\;\mathrm{MPa})$$

In Equation (2), $f_{co}^{’}$ is the unconfined insulation material strength, $f_{l}^{’}$ is the effective lateral confined stress of CFRP, and ϕ is the internal friction angle of insulation material. In Equation (3), $k_{c}$ is the coefficient of the section shape, *n* is the number of the layers of CFRP, *t* is the thickness of CFRP per layer, $E_{cf}$ is the elastic modulus of CFRP, $\varepsilon_{cf}$ is the ultimate strain of CFRP measured from the strain gauge in the compression test ($\varepsilon_{cf}$=1 %, obtained from the compression test), and *D* is the diameter of the cylinder. In Equation (2), the internal friction angle depends on the insulation material strength, and it can be expressed as the linear relationship of insulation material strength as shown in Equation (4), where *a* and *b* are coefficients to be determined by regression analysis.

(4) ϕ=a0+10(fco’b)≤450 (Unit: degree)

**Figure 10:**
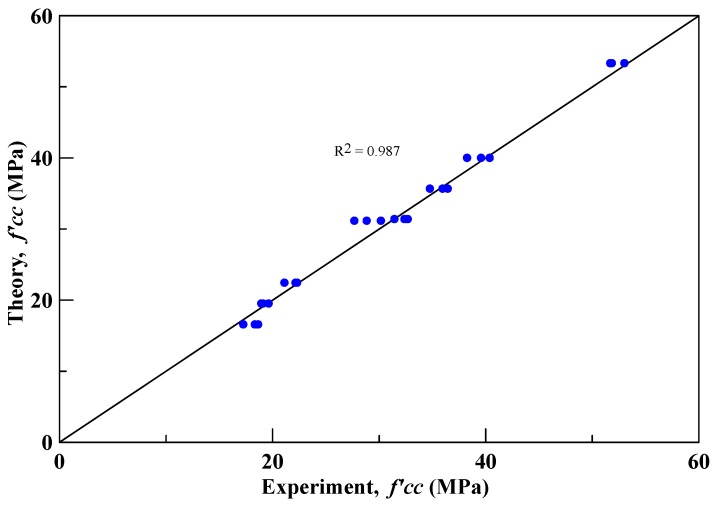
The relationship diagram between the experimental and proposed theoretical strengths.

From the regression analysis, this study obtained *a* = 16 and *b* = 7. The experimental and proposed theoretical compressive strengths of the insulation material are shown in Figure 10; and the square of the correlation coefficient ($R^{2}$) is 0.987. As shown in Table 7, the average absolute error of the compressive strength between experimental data and the proposed theoretical formula is 3.33%. The proposed formula can accurately predict the compressive strengths of the specimen with the confined insulation material.

## 6. Verification Test of the Proposed Strengthening Method

In this section, the verification test of the strengthening method for the high-temperature-used pipeline was performed. Three different thicknesses and three different perlite ratios of C-shape precast insulating materials were used in the test. 

### 6.1. Experimental Program

**Figure 11:**
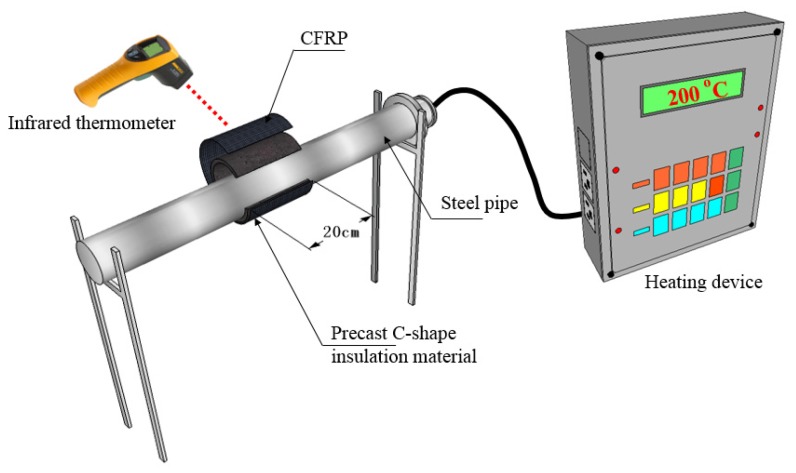
Illustration of experimental setup for high-temperature pipeline strengthening method.

A copper-headed heating rod was put into a stainless steel tube with an outer diameter of 11.4 cm and a length of 100 cm, and the stainless steel tube was heated by a heating device to simulate high-temperature pipeline. The temperature record of the CFRP on its surface was measured by an infrared thermometer. The experimental equipment installation for the high-temperature pipeline maintenance method is shown in Figure 11. 

The component design of C-shaped precast insulating materials included the following step: Three different perlite ratios, 8%, 15%, and 25% with a fixed thickness of 25 mm; and three different thicknesses, 15, 25, and 35 mm with a fixed perlite ratio 25%. A total of five specimens are classified as shown in Table 8. 

### 6.2. Experimental Results and Observations

The experiment aimed for understanding the effect of the different additional perlite ratios on heat insulation. For the fixed thickness of 25 mm, three different perlite ratios of C-shaped precast insulating materials were planned, namely specimens P08-25, P15-25, and P25-25.

**Figure 12:**
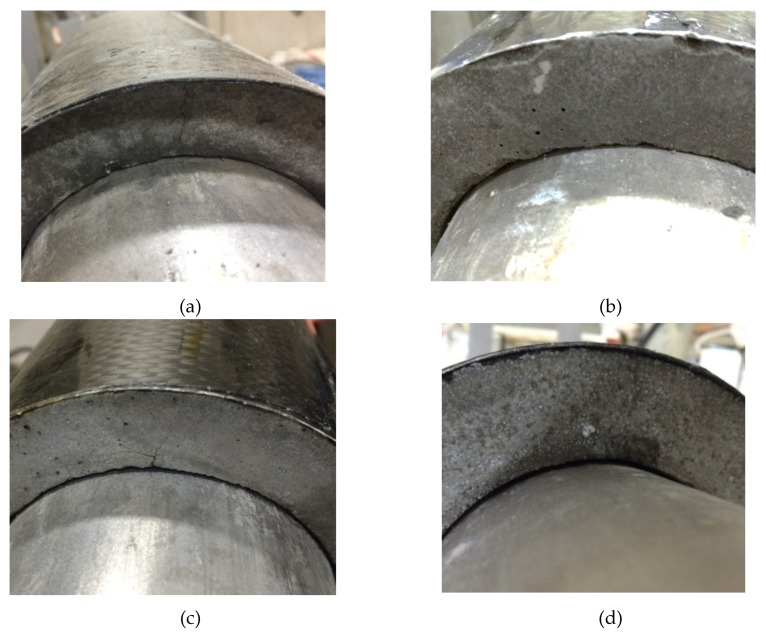
Few hairline cracks were detected in the C-shaped insulation materials after heating. (**a**) Specimen P08-25; (**b**) Specimen P15-25; (**c**) Specimen P25-25; (**d**) Specimen P25-35.

Specimen P25-25 was chosen for the first experiment, specimen P25-25 had the content of 25% perlite with the C-shape precast insulation material of 25 mm-thick. When the steel pipe was heated up to 200 ℃ for 17 hours and the heating system stay in steady state; the surface temperature of CFRP is about 85 ℃. The temperature difference between the steel pipe and CFRP surface is 115℃ due to the insulation effect of C-shaped insulation material. The C-shaped insulation material was removed after the steel tube cooled to room temperature; few hairline cracks were observed in the specimen P25-25 owing to the thermal impact. However, CFRP composite material was completely undamaged; the observation image of the specimen P25-25 as shown in Figure 12 although the fracture texture only occurs with few hairline cracks after the thermal experiment. As seen in Figure 12a–c, specimen P08-25 and P15-25, the experimental results were similar to the results of specimen P25-25, except for the temperatures on the CFRP surface. The reduced temperatures of the specimens with different perlite ratios are shown in Table 9. As seen from experimental results of a greater reduced temperature value, the high temperature using pipelines decreased thermal damage in a higher composition of the perlite ratio of the C-shaped insulation materials and, therefore, it had a stronger reinforced capability. 

Three different thicknesses of C-shaped precast insulating materials were investigated, namely specimens P25-15, P25-25, and P25-35, for the fixed perlite ratio. When the steel pipe was heated up to 200℃ for 17 hours, the surface temperature of CFRP were about 104 °C and 75 °C for specimens GP25-P15 and GP25-35, respectively. Similarly, few hairline cracks were detected in the specimen P25-15 and GP25-35 due to thermal damage, the experimental results were similar to the results of specimen P25-25 after a thermal attack, except the temperatures on the CFRP surface, shown in Figure 12c,d. The measured temperatures of the specimens with different thicknesses are shown in Table 10. As seen from experimental results in Table 10, the thickness of the C-shaped insulation materials increased the reduced temperature of the insulation materials.

## 7. Conclusions

Based on the results of this study, the following conclusions can be drawn:

1. The compressive strength of the specimen mixed with perlite powder decreased from 35.6 MPa to 1.73 MPa, and thermal conductivity of the specimen decreased from 0.428 W/(m°C) to 0.295 W/m℃ by increasing the perlite powder ratio from 0% to 25%. Similarly, the compressive strength of the specimen mixed with vermiculite powder decreased from 35.6 MPa to 3.5 MPa; and thermal conductivity of the specimen decreased from 0.428 W/(m°C) to 0.344 W/(m°C) by increasing the amount of the additional vermiculite ratios from 0% to 25%. Therefore, as the perlite or vermiculite powder ratio increases, the compressive strength and thermal conductivity of the specimen will decrease.
2. The compressive strength of the specimens with different perlite ratio increased by 258%–927% after reinforced using 1-layer CFRP composite material, and increased by 480%–1541% after reinforced using 2-layer CFRP composite material.
3. In comparing the experimental results of the 36 specimens, the average absolute errors of the peak strength estimation of the proposed formula were less than 3.33%. The proposed “peak strength formula” can effectively predict the peak strength of the perlite powder mixed with MPC cylindrical specimen confined by CFRP composite material.
4. In the strengthening method for the high-temperature pipeline test, when the perlite ratios in the C-shaped insulation materials increased from 8% to 25%, the reduction in temperature increased from 102 °C to 115 °C. The higher perlite ratio added in the C-shaped insulation materials, the larger the reduction in temperature of the insulation materials occurred.
5. When the thickness of the C-shaped insulation materials increased from 15 mm to 35 mm, the reduced temperature of the specimen increased from 96 °C to 125 °C. The thickness of the C-shaped insulation materials increased and led to a greater reduced temperature of the insulation materials.
6. From the verification test of the strengthening method for damaged high-temperature pipeline, it was shown that the proposed strengthening method is feasibable.

## Figures and Tables

**Table 1:** The notation definition of the specimen with various additional amounts of powders.

Name	The Percentage of the Additional Powder (%)
C	0
PC	5; 10; 15; 20; 25
PT	5; 10; 15; 20; 25
T	0
VC	5; 10; 15; 20; 25
VT	5; 10; 15; 20; 25

**Table 2:** Material properties of the carbon fiber sheet and epoxy resin.

**Carbon Fiber Sheet**	**Material Specification**	**FAW 300 (g/m^2^)**
	Young’s modulus, *E_cf_*	250 (GPa)
	Tensile strength	4.9 (GPa)
	Thickness	0.16 (mm/layer)
	Ultimate strain	0.02
Epoxy resin	Viscosity (25 °C)	1823 (cps)
	Young’s Modulus	3.5 GPa
	Tensile Strength	52.2 (MPa)
	Tensile adhesive strength	10.5 MPa

**Table 3:** The average 3-day compressive strength of the classfied specimens.

Specimen	Average Compression Strength (MPa)	Specimen	Average Compression Strength (MPa)
C0	35.60	-	-
PC05	18.20	VC05	21.67
PC10	7.85	VC10	14.38
PC15	3.81	VC15	9.02
PC20	2.39	VC20	5.2
PC25	1.73	VC25	3.5

**Table 4:** The average thermal conductivity coefficient of the specimens.

Specimen	Average Thermal Conductivity Coefficient (W/(m°C))	Specimen	Average Thermal Conductivity Coefficient (W/(m°C))
T0	0.428	-	-
PT05	0.411	VT05	0.422
PT10	0.374	VT10	0.412
PT15	0.346	VT15	0.393
PT20	0.322	VT20	0.365
PT25	0.295	VT25	0.344

**Table 5:** The design parameters and specimen notation of 36 concrete cylinders.

Specimen	Diameter and Height of Cylinder	Unconfined	1-Layer CFRP	2-Layer CFRP
P08	φ10 cm × 20 cm	3	3	3
P15	φ10 cm × 20 cm	3	3	3
P20	φ10 cm × 20 cm	3	3	3
P25	φ10 cm × 20 cm	3	3	3

**Table 6:** The compressive strength of insulation materials after applying by CFRP.

Specimen	CFRP, No. of Layer	Compressive Strength (MPa)	Average compressive Strength (MPa)	Increase Percentage (%)
P08	0	9.44	8.99	-
		8.73		
		8.80		
P08C1	1	31.43	32.16	258
		32.68		
		32.37		
P08C2	2	53.01	52.18	480
		51.83		
		51.69		
P15	0	4.92	4.88	-
		4.70		
		5.02		
P15C1	1	22.30	21.85	348
		21.11		
		22.15		
P15C2	2	38.25	39.39	707
		40.37		
		39.56		
P20	0	3.47	3.36	-
		3.16		
		3.55		
P20C1	1	19.63	19.22	472
		18.94		
		19.09		
P20C2	2	34.76	35.72	963
		36.44		
		35.95		
P25	0	1.83	1.76	-
		1.75		
		1.71		
P25C1	1	18.34	18.08	927
		17.25		
		18.64		
P25C2	2	30.16	28.89	1541
		28.83		
		27.67		

**Table 7:** The compressive strength error analysis between the experiment and proposed formula.

Specimen	$f_{c}^{’}$ (MPa)	$f_{l}$ (MPa)	Experiment, $f_{cc}^{’}$ (MPa)	Proposed Formula, $f_{cc}^{’}$ (MPa)	Error (%)
P08C1	8.99	7.67	32.16	42.11	-3.01
P08C2	8.99	15.33	52.18	53.34	2.23
P15C1	4.88	7.67	21.85	22.45	2.74
P15C2	4.88	15.33	39.39	40.02	1.59
P20C1	3.36	7.67	19.22	19.52	0.02
P20C2	3.36	15.33	35.72	35.69	-0.09
P25C1	1.76	7.67	18.08	16.58	-8.27
P25C2	1.76	15.33	28.09	31.41	8.72
Average absolute error (%)					3.33

**Table 8:** The specimen notation scheme with different thicknesses.

Thickness (mm)	15	25	35
P08	-	P08-25	-
P15	-	P15-25	-
P25	P25-15	P25-25	P25-35

**Table 9:** The measured temperatures and notation of different perlite ratios specimens.

Specimen	Steel Pipe Temperature (°C)	CFRP Temperature (°C)	Reduced Temperature (°C)
P08-25	200	98	102
P15-25	200	91	109
P25-25	200	85	115

**Table 10:** The measured CFRP temperatures of different thickness specimens.

Specimen	Steel Pipe Temperature (°C)	CFRP Temperature (°C)	Reduced Temperature (°C)
P25-15	200	104	96
P25-25	200	85	115
P25-35	200	75	125
